# Betulinic Acid Inhibits Collagen-Induced Platelet Adhesion and Activation Through Inhibition of Spleen Tyrosine Kinase (SYK) Phosphorylation

**DOI:** 10.7759/cureus.93455

**Published:** 2025-09-28

**Authors:** Hariprasath Ragupathy, Saba Anjum, Ayana E Kuttapan, Glenda Maria, William R Surin

**Affiliations:** 1 Microbiology and Cell Biology, Indian Institute of Science, Bengaluru, IND

**Keywords:** betulinic acid, collagen, flow cytometry, platelets, syk phosphorylation

## Abstract

Betulinic acid is a pentacyclic triterpenoid and is found in various plant species. Betulinic acid has been shown to exhibit several biological activities, such as antiviral, anticancer, immunomodulatory, anti-inflammatory, antimicrobial, anti-diabetic, anti-parasitic, and anti-HIV properties. Additionally, betulinic acid has shown antiplatelet effects against thrombin receptor activator peptide (TRAP), adenosine diphosphate (ADP), and arachidonic acid (AA)-induced platelet activation and aggregation. However, its effect on collagen-induced platelet activation and signaling pathways is not yet known. Therefore, we investigated the effect of betulinic acid on collagen-induced platelet adhesion and activation. Platelets were isolated from mice and labeled with CD41 PE-Cy7 at 37 °C for one hour and induced with various agonists to determine the platelet activation profile against different platelet agonists. Further, the concentration-dependent effect of betulinic acid (100 µM, 300 µM, 500 µM, or 1 mM) against collagen-induced platelet activation was assessed by flow cytometry. Moreover, platelets were labeled with CD41 PE-Cy7 and phospho-spleen tyrosine kinase (SYK) PE at 37 °C for one hour and incubated with betulinic acid (500 µM) or cytochalasin D (1 µg/mL) for 20 minutes, followed by the addition of collagen (25 µg/mL). Thereafter, samples were analyzed by flow cytometer (BD FACSCanto^TM^ II) to determine their platelet activation and SYK phosphorylation. Further, washed platelets labeled with CD41 PE-Cy7 were incubated with phalloidin-fluorescein isothiocyanate (FITC) and pre-treated with either betulinic acid (500 µM) or cytochalasin D (1 µg/mL), followed by 30 minutes of incubation on a collagen-coated cover slip for platelet adhesion studies by confocal microscopy. Analysis of data was performed by one-way ANOVA, followed by post-hoc Dunnett’s test concerning the control group using GraphPad Prism statistical software (GraphPad Software, San Diego, CA). All the experiments were performed in duplicates of ≥3 independent experiments. All the data were reported as mean ± SEM in all the groups. P < 0.5 was considered to be statistically significant. Betulinic acid inhibited the collagen-induced platelet activation in a concentration-dependent manner at 500 µM and 1 mM (6091 ± 901 vs 3569 + 291, 6091 ± 901* *vs* *3305 + 623)and decreased the SYK phosphorylation by inhibiting protein tyrosine kinase SYK in flow cytometry studies. Further, it inhibited the platelet adhesion on the collagen-coated surface in imaging studies. Betulinic acid inhibited the collagen-induced platelet activation and reduced SYK phosphorylation from 82.1% to 49.8%. It has shown better inhibition than cytochalasin D (60.2%). Moreover, betulinic acid exhibited a platelet inhibitory effect on platelet adhesion on collagen-coated surfaces. These studies show that betulinic acid inhibits collagen-induced platelet activation through a reduction in SYK phosphorylation, corroborating the antiplatelet activities of betulinic acid.

## Introduction

Betulinic acid is a pentacyclic triterpenoid, obtained from numerous plant species such as *Orthosiphon stamineus*, *Vitex negundo*, *Eucalyptus camaldulensis*, and *Tetracentron sinense* [1]. Betulinic acid is produced by betulin oxidation and is widely distributed in nature. It has been shown to exhibit several biological activities, such as antiviral, anticancer, immunomodulatory, anti-inflammatory, antimicrobial, antidiabetic, antiparasitic, and anti-HIV [1-3]. Betulinic acid has been extensively studied and was found to inhibit the proliferation of cancer cells. Additionally, it induced apoptosis of cancer cell lines, such as the colon, breast, melanoma, prostate, leukemia, and brain. Further, various in vivo investigations have proven that betulinic acid is relatively non-toxic for healthy cells and has a high safety margin, as systemic adverse effects were not observed at the studied dosage range [1,2,4]. Moreover, betulinic acid has the potential to modulate several immune system cell types, including macrophages and lymphocytes, and it has been demonstrated in several models of inflammation to have an anti-inflammatory effect. Many studies have shown the promising immunomodulatory activity of betulinic acid [3].

There were many studies conducted to determine the pharmacological activity of betulinic acid. However, only a few studies confirmed the antithrombotic effect of betulinic acid. In an earlier study, betulinic acid and betulin were used dose-dependently to evaluate the inhibitory activity against thrombin receptor activator peptide-14 (TRAP), adenosine 5’-diphosphate, and arachidonic acid (AA)-induced platelet activation, and it was observed that betulinic acid inhibited platelet aggregation induced by AA and TRAP [5]. However, the effect of betulinic acid on collagen-induced platelet adhesion and activation is yet to be investigated.

Therefore, we assessed the effect of betulinic acid on collagen-induced platelet adhesion, platelet activation, and spleen tyrosine kinase (SYK) phosphorylation, as SYK phosphorylation has been known to play a crucial role in relaying intracellular signaling in platelets.

## Materials and methods

Materials

Collagen type I from rat tail, collagen from calf skin, thrombin from human plasma, thromboxane A2 analog U46619, calcium ionophore A23187, Phorbol 12-myristate 13-acetate (PMA), adenosine 5′-diphosphate (ADP), apyrase, HEPES buffer, betulinic acid, phalloidin-fluorescein isothiocyanate (FITC), and cytochalasin D were procured from Sigma-Aldrich Chemicals Inc. (Burlington, MA). Phospho-ZAP70/SYK(Tyr319, Tyr352) monoclonal antibody (n3kobu5), PE and CD41a monoclonal antibody (eBioMWReg30 (MWReg30)), and PE-Cy7 were from Thermofisher (Waltham, MA). All the other chemicals used were of analytical grade.

Animals

Male BALB/c mice (20-25 g), obtained from the Central Animal Facility, Indian Institute of Science, Bangalore, India, were caged and acclimatized in a pathogen-free environment at a room temperature of 24°C ± 0.5°C, 12 hours light/dark cycle with relative humidity (55 + 10%), and were provided chow pellets and water ad libitum. All the experiments were performed in accordance with the guidelines of the Committee for the Purpose of Control and Supervision of Experiments on Animals, with the prior approval of the Institute Animal Ethics Committee of the Indian Institute of Science, Bangalore (CAF/Ethics/679/2019).

Washed platelet preparation

Healthy BALB/C mice were utilized for the experiment, and it was anesthetized with isoflurane. The blood was drawn from the mice by cardiac puncture using a syringe containing 3.8% tri-sodium citrate (anticoagulant) in a ratio of 1:9. The collected blood was centrifuged at 275 g for 15 minutes at 20°C to obtain platelet-rich plasma (PRP) and transferred into a new Eppendorf tube containing 1 μL aspirin and 10 μL apyrase and incubated at 37°C for 30 minutes. After the addition of 5 μL of 5 mM Na. EDTA (ethylenedinitrilo tetraacetic acid disodium salt dihydrate), it was centrifuged at 800 g for 15 minutes at 37°C. The cell pellet was collected and suspended in 1 mL of buffer A (20 mM HEPES, 138 mM NaCl, 2.9 mM KCl, 1 mM MgCl2, 0.36 mM NaH2PO4, 1 mM EGTA, supplemented with 5 mM glucose, and 0.6 ADPase units/mL of apyrase, pH 6.2) and centrifuged at 800 g for 20 minutes. The platelets were further suspended in buffer B (pH 7.4), identical to buffer A, except for the absence of EGTA and apyrase, and used for further analysis [6].

Effect of various agonists on platelet activation

We evaluated the effect of various platelet agonists on platelet activation. The washed platelets were aliquoted into different vials and incubated with CD41 PE-Cy7 at 37°C for one hour. Platelet activation was induced by collagen from rat tail (25 µg/mL), collagen from calf skin (25 µg/mL), thrombin (0.5 U/mL), thromboxane A2 analog U46619 (5 µM), calcium ionophore A23187 (5 µM), Phorbol 12-myristate 13-acetate (3 µM), or ADP (25 µM) individually. The samples were analyzed by flow cytometry at excitation and emission of 566 and 778 nm, respectively (BD FACSCanto^TM^ II; BD Biosciences, Inc., Franklin Lakes, NJ). At least 100,000 events per sample were acquired. Data were analyzed with BD FACSDiva™ software [6].

Effect of betulinic acid on collagen-induced platelet activation by flow cytometry analysis

The washed platelets were incubated with CD41 PE-Cy7 in the dark and incubated at 37°C for one hour, followed by incubation with increasing concentrations of betulinic acid (100 µM, 300 µM, 500 µM, or 1 mM) for 20 minutes, followed by activation with rat tail collagen (25 μg/mL) to determine the concentration-dependent effect of betulinic acid on collagen-induced platelet activation. The samples were analyzed at excitation and emission of 566 and 778 nm, respectively (BD FACSCanto^TM^ II; BD Biosciences, Inc). At least 100,000 events per sample were acquired. Data were analyzed with BD FACSDiva™ software [6].

Effect of betulinic acid on SYK phosphorylation

The washed platelets were aliquoted into different vials. The Phospho-SYK PE and CD41, PE-Cy7 (Thermofisher, Inc.) were added in the dark and incubated at 37°C for one hour. Thereafter, the platelets were incubated with inhibitors cytochalasin D (1 µg/mL) or betulinic acid (500 µM) for 20 minutes. Platelet activation was assessed by adding collagen (25 μg/mL). The samples were analyzed by a flow cytometer at the fluorescence excitation and emission of 566 nm and 574 nm for Phospho-SYK PE and at excitation and emission of 566 nm and 778 nm for CD41 P-Cy7 (BD FACSCantoTM II, BD Biosciences, Inc). At least 100,000 events per sample were acquired. Data were analyzed with BD FACSDiva™ software [6].

Effect of betulinic acid on platelet adhesion by confocal microscopy

The 0.1 % (w/v) stock solution of collagen (rat tail, type I) was prepared in 0.1 M acetic acid. The working solution was prepared by diluting 10-fold to 0.01%. About 100 µL of collagen solution was coated on the glass cover slips and kept at 4°C overnight. Then, the slides were washed with phosphate buffer saline (PBS) and blocked using 0.5% heat-denatured bovine serum albumin in PBS for one hour at room temperature, followed by washing in PBS. The washed platelets were incubated with CD41 PE-Cy7 for one hour and with betulinic acid (500 µM) and cytochalasin D (1 µg/ml) for 20 minutes at 37°C. Then, it was transferred to the cover slips and incubated at 37°C for one hour and in UV for 20 minutes. Excess platelets were removed by washing with PBS, and the adherent platelets were fixed with 4% (w/v) paraformaldehyde for 10 minutes at room temperature. This solution was removed, and coverslips were washed three times with PBS before adding 0.1% (v/v) Triton-X-100 to permeabilize the cells. After removing Triton-X, platelets were washed with PBS and stained with phalloidin-FITC (50 µg/mL) and incubated for 30 minutes in the dark at room temperature. Coverslips were mounted onto microscope glass slides and imaged using Leica TCS SP8 Confocal Microscope under a 63× oil-immersion lens. The excitation and emission ranges of phalloidin-FITC are 488 nm and 520 nm, respectively. The CD41 PE-Cy7 was observed at excitation and emission of 566 and 778 nm, respectively [7,8].

Statistical analysis

All the experiments were performed in duplicates of ≥3 independent experiments. Simultaneously, analysis of data was performed by one‑way ANOVA, followed by post hoc Dunnett’s test concerning the control group using GraphPad Prism statistical software (GraphPad Software, San Diego, CA). All the data were reported as mean ± SEM in all the groups. P < 0.5 was considered to be statistically significant.

## Results

Platelet activation with various agonists in flow cytometry

**Figure 1:**
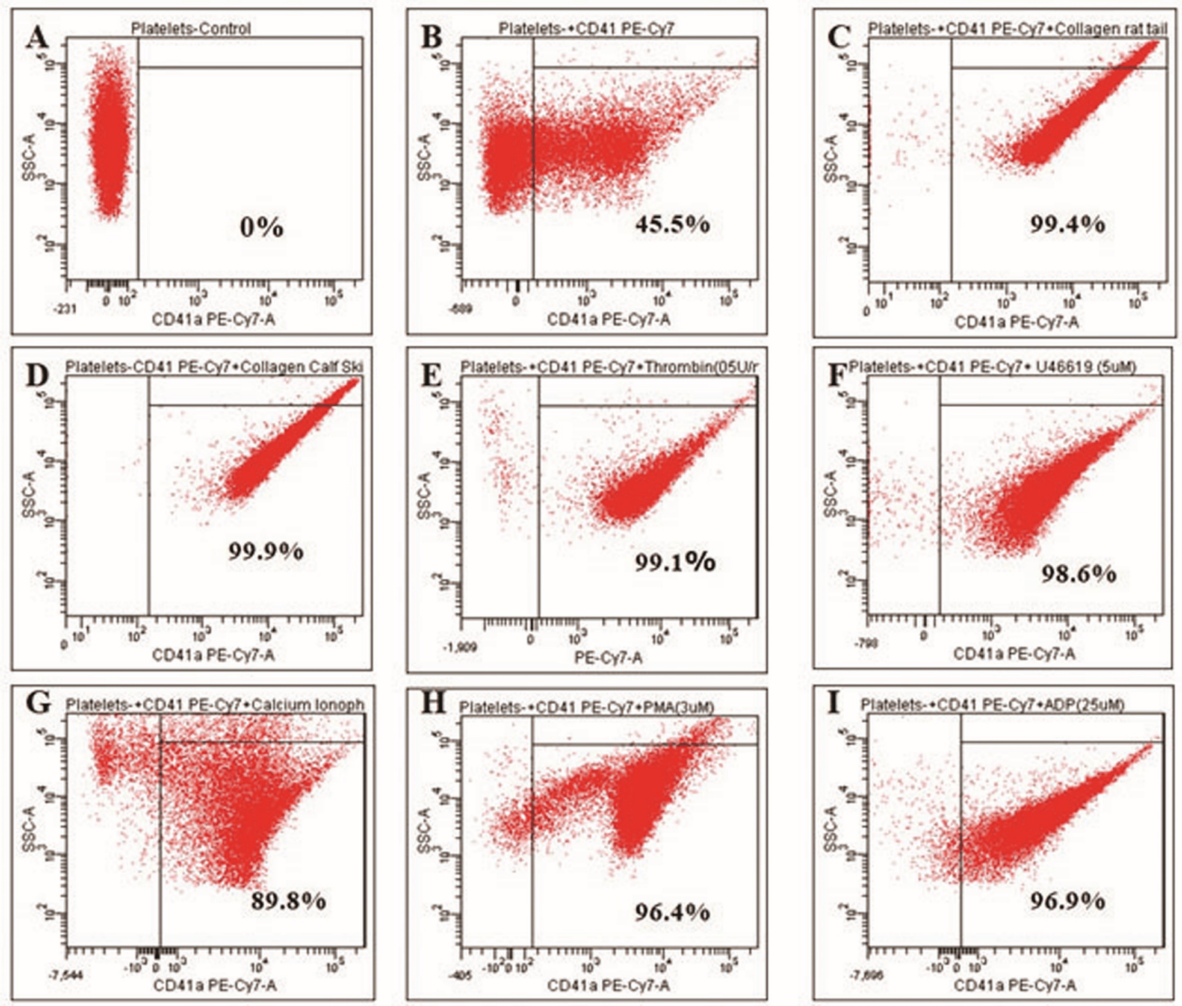
Effect of various agonists on platelet activation (A) Platelets alone. (B) Platelets + CD41 PE-Cy7. (C) Platelets + CD41 PE-Cy7+ Rat tail collagen (25 µg/mL). (D) Platelets + CD41 PE-Cy7+ Calf skin collagen (25 µg/mL). (E) Platelets + CD41 PE-Cy7+ thrombin (0.5 U/mL). (F) Platelets + CD41 PE-Cy7+ U46619 (5 µM). (G) Platelets + CD41 PE-Cy7+ A23187 (5 µM). (H) Platelets + CD41 PE-Cy7+ PMA (3 µM). (I) Platelets + CD41 PE-Cy7+ ADP (25 µM)

The platelets were isolated from the mice and were induced with different agonists to optimize their concentration to induce platelet activation. Platelet samples were activated with collagen from rat tail (25 µg/mL), collagen from calf skin (25 µg/mL), thrombin (0.5 U/mL), thromboxane A2 analog U46619 (5 µM), calcium ionophore A23187 (5 µM), Phorbol 12-myristate 13-acetate, PMA (3 µM), and ADP (25 µM). Platelet activation was assessed by flow cytometry by measuring the expression of CD41, which is conjugated with PE-Cy7. The more platelet activation, the more the expression of CD41 PE-Cy7 (Figure 1). Platelet activation exhibited by thrombin (0.5 U/mL), thromboxane A2 analog U46619 (5 µM), calcium ionophore A23187 (5 µM), Phorbol 12-myristate 13-acetate (3 µM), and ADP (25 µM) was 99.1%, 98.6%, 89.8%, 96.4%, and 96.9%, respectively. Collagen from rat tail (25 µg/mL) and collagen from calf skin (25 µg/mL) exhibited 99.4% and 99.9% platelet activation, respectively. Therefore, collagen from rat tail at a concentration of 25 µg/mL was used for subsequent studies.

**Figure 2:**
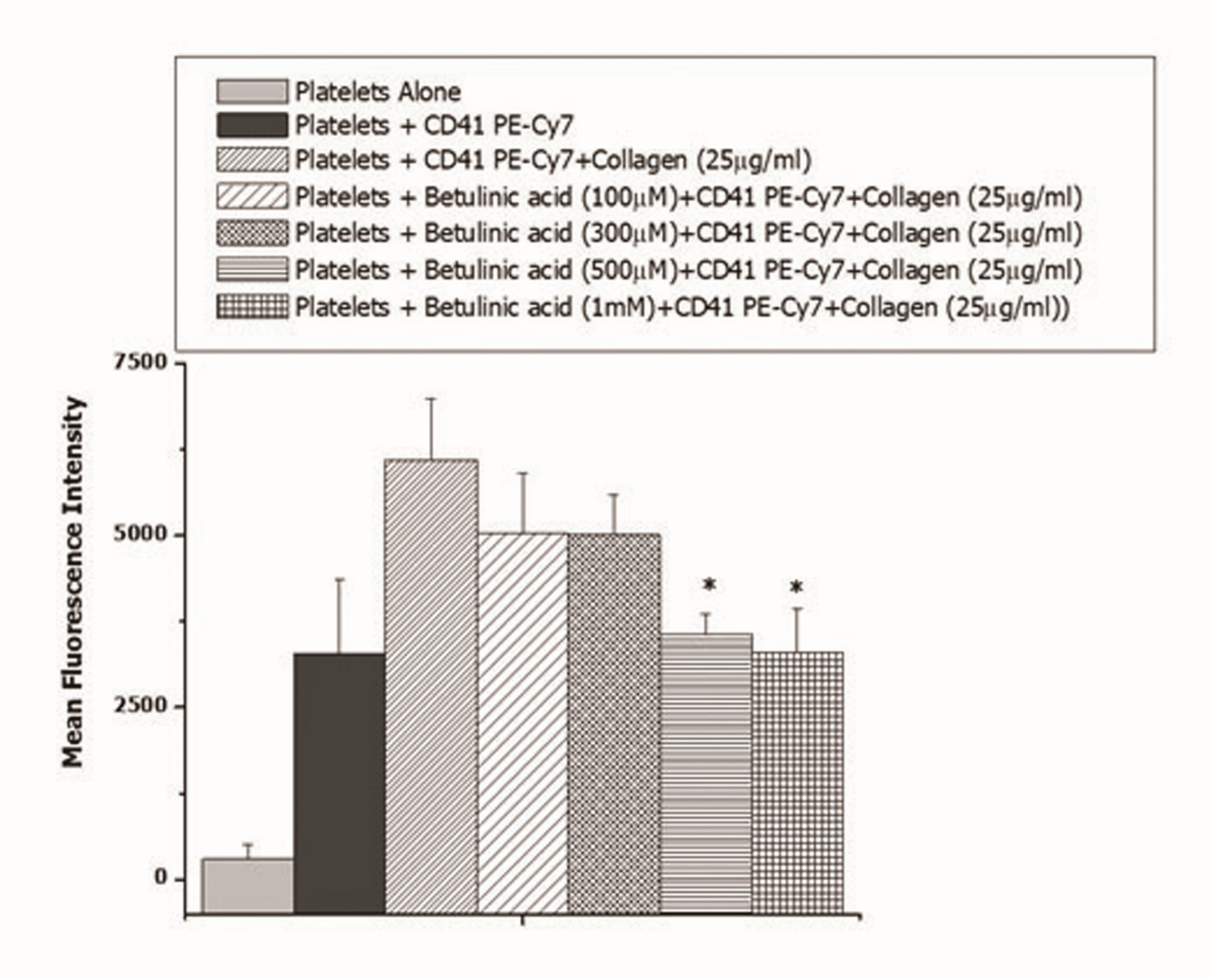
Effect of betulinic acid (100 µM to 1 mM) on collagen-induced platelet activation For *: P < 0.05

Further, we assessed the effect of betulinic acid on collagen-induced platelet activation in a concentration-dependent manner (Figure 2). Betulinic acid was found to inhibit collagen-induced platelet activation at 500 µM and 1 mM.

**Figure 3:**
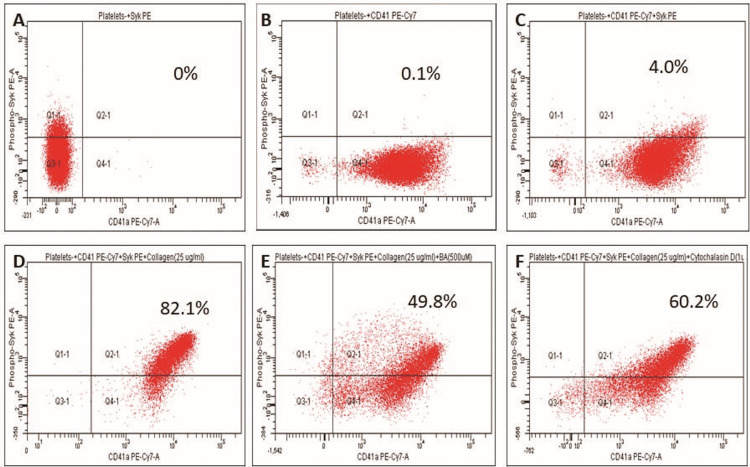
Effect of betulinic acid (500 µM) on collagen-induced SYK phosphorylation in the platelets (A) Platelets alone. (B) Platelets + CD41 PE-Cy7. (C) Platelets + CD41 PE-Cy7+Phospho SYK PE. (D) Platelets + CD41 PE-Cy7+ Phospho SYK PE +Rat tail collagen (25 µg/mL). (E) Platelets + CD41 PE-Cy7+ Phospho SYK PE +betulinic acid (500 µM)+Rat tail collagen (25 µg/mL). (F) Platelets + CD41 PE-Cy7+ Phospho SYK PE +Cytochalasin D (1µg/ml)+Rat tail collagen (25 µg/mL)

Thereafter, we assessed the effect of betulinic acid on SYK phosphorylation in collagen-induced platelet activation (Figure 3). There was a significant increase in collagen-induced SYK phosphorylation in platelets from that of the control sample (4.0% vs 82.1%) (Figure 3). Betulinic acid significantly decreased collagen-induced SYK phosphorylation (82.1% vs 49.8%) (Figure 3). Further, cytochalasin D decreased collagen-induced SYK phosphorylation (82.1% vs 60.2%). The percent inhibition exhibited by betulinic acid was higher compared to the positive control, cytochalasin D (49.8% vs 60.2%) (Figure 3).

Platelet adhesion by confocal microscopy

**Figure 4:**
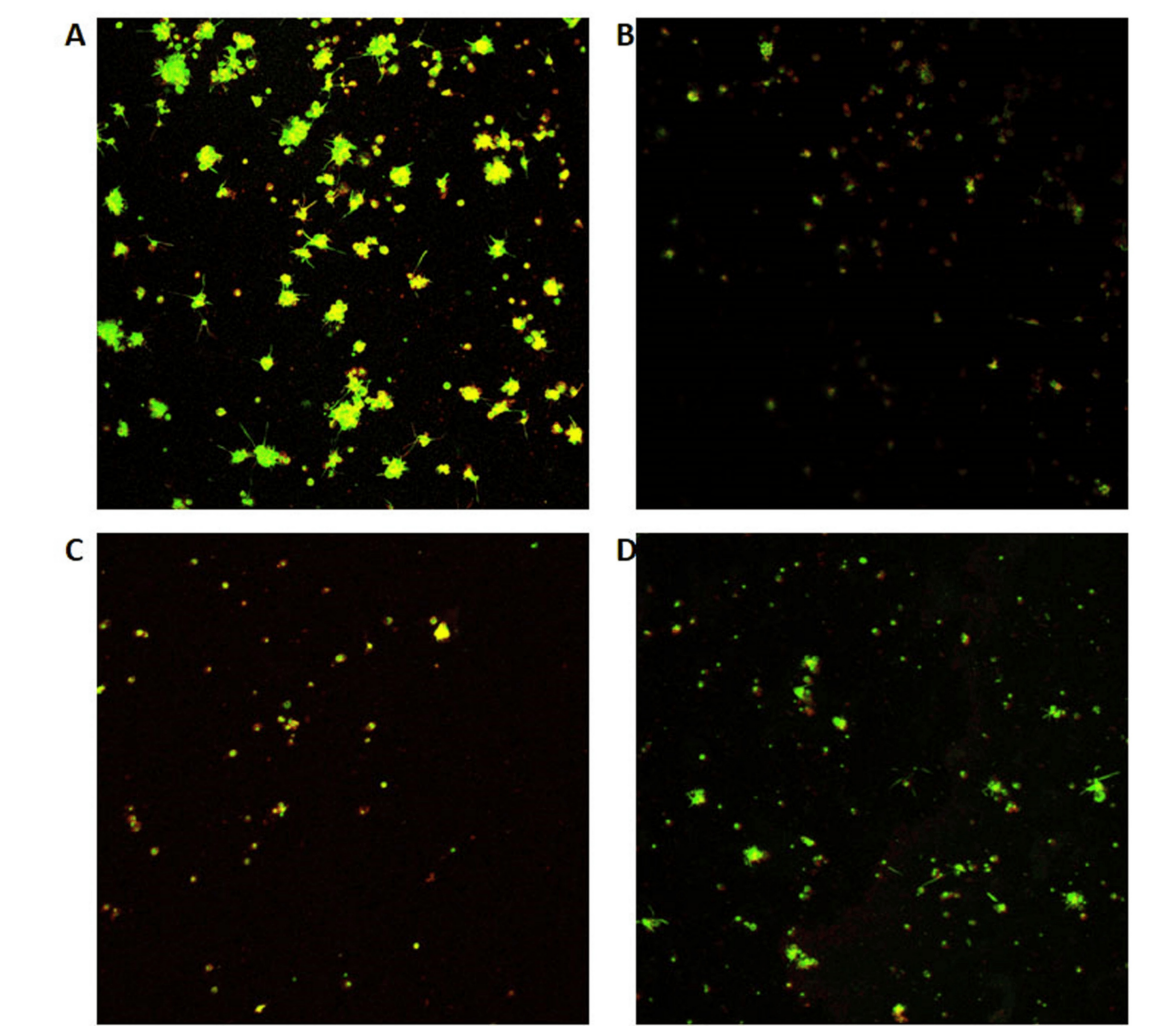
Effect of betulinic acid on platelet adhesion on the collagen-coated surface (A) Platelets labeled with CD41 PE-Cy7 and phalloidin-FITC on collagen-coated surface. (B) Platelets labeled with CD41 PE-Cy7 and phalloidin-FITC on blank surface. (C) Cytochalasin D (1 µg/mL) pre-treated platelets labeled with CD41 PE-Cy7 and phalloidin-FITC on collagen-coated surface. (D) Betulinic acid (500 µM) pre-treated platelets labeled with CD41 PE-Cy7 and phalloidin-FITC on the collagen-coated surface

The interaction of fluorophores with resting and active mouse platelets was confirmed using confocal microscopy. The resting platelets with fluorophores on the collagen-coated surface were treated with the positive control cytochalasin D (1 µg/mL) and with our target compound, betulinic acid (500 µM). The phalloidin-FITC was used, which stains the platelets and quantifies F-actin content. The results of the confocal images are given in Figure 4. The aggregated and resting platelets were unspread, with filopodia and lamellipodia, indicating the actin protrusion formation. 

In contrast, platelets with inhibitors were round and unspread and lacked filopodia or lamellipodia on a collagen-coated surface under identical spreading conditions. Compared to cytochalasin D, platelets treated with phytochemical, betulinic acid, have shown better inactivation, which has shown a change in platelet shape from discoid to round under a confocal microscope that demonstrates few or no filopodia and lacks lamellipodial protrusions. Therefore, our study suggests that betulinic acid played an important role in inhibiting platelet adhesion and activation.

## Discussion

Various synthetic and natural substances have been reported to inhibit platelet-collagen interactions. Our current study provides the first evidence of the inhibitory activity of betulinic acid on SYK phosphorylation in collagen-induced platelet activation by inhibiting the protein tyrosine kinase SYK. Many studies conducted by various researchers have investigated the effect of betulinic acid on human platelets by using various platelet agonists. Betulinic acid has been reported to inhibit platelet activation and aggregation induced by arachidonic acid, TRAP, and thrombin, with inhibitory activity observed at 440 μM [5]. However, the effect of betulinic acid on collagen-induced platelet activation has not yet been investigated. Collagen is mostly present on the sub-endothelial surface and gets exposed during vascular injury. It acts as an adhesive surface and is a very strong activator of platelets. The two most well-studied and important receptors identified on the surface for collagen-platelet interactions are glycoprotein VI (GPVI) and glycoprotein Ia/IIa (GPIa/IIa or integrin α2β1). Glycoprotein VI is one of the G-protein-coupled receptors and a collagen receptor inextricably linked to the FcRγ. The binding of collagen to the GPVI receptor leads to the activation of GPVI, followed by the phosphorylation of FcRγ on the tyrosine residue of its ITAM. This is aided by Src family kinases (SFK) and SYK. Spleen tyrosine kinase (SYK) is a nonreceptor tyrosine kinase with a 72-kDa molecular weight, and it has been linked to various signaling cascades in platelets [9,10]. The phosphorylation of SYK relies on tyrosine kinase pathway receptors such as glycoprotein VI (GPVI)/Fc receptor, the hemITAM-containing C-type lectin-like receptor-2 (CLEC-2), and αIIbβ3 integrin. This complex probably activates SYK through both conventional ITAM‑mediated SYK activation pathways and by binding the integrin β‑chain to the amino‑terminal SH2 domain of SYK in a phosphotyrosine‑independent manner. All three cases of SYK activation lead to platelet activation through the SH2 domain-containing leukocyte protein 76 (SLP76). Additionally, SYK phosphorylates and activates the downstream mediators following PLCγ and 1,2-diacylglycerol-activated PKC and inositol (1,4,5)-triphosphate-liberated Ca2^+^. In turn, these molecules engage other signal transduction pathways. This will result in platelet activation and aggregation [9-11]. Previous studies have demonstrated that blocking SYK reduced platelet activation and aggregation properties for collagen peptides with or without a (GPO)n sequence in whole blood underflow [12]. Therefore, Jooss et al. [12] focused on determining the thrombogenic activity of various collagen peptides and (fibrillar) type I and III collagens in the SYK-mediated pathway by using SYK inhibitor PRT-060318. Finally, they observed that SYK inhibition reduced the collagen-induced platelet activation and aggregation characteristics [12,13]. Based on previous reports, protein SYK plays a role in the platelet signaling pathway. Therefore, inhibition of protein SYK could lead to the inactivation of downstream events in platelet signaling. Therefore, we evaluated the potential activity of betulinic acid against the SYK protein in our study. The expression of protein tyrosine kinase SYK and collagen-induced platelet activation were analyzed in flow cytometry. Flow cytometry thus provides a numerical technique for assessing platelet activity that is both objective and quantitative. In previous studies, phycoerythrin-labeled monoclonal antibodies such as CD41 PE, CD63 PE, CD62P PE, and CD42b PE are all platelet-specific markers used in platelet studies for flow cytometry analysis [14]. In the present study, we used CD41 PE-Cy7 antibody to assess collagen-induced platelet activation and SYK phosphorylation, which is the α subunit of the CD41/CD61 complex (GPllb-llla) and phospho-SYK PE. Betulinic acid reduced the binding of CD 41 PE Cy-7 and SYK phosphorylation on platelets, confirming the antiplatelet activity of betulinic acid.

Phalloidin-FITC has been used in confocal microscopy analysis to study platelet adhesion and activation on collagen-coated surfaces. Phalloidin is the most commonly used dye that stains the F-actin content of the platelets [15]. Betulinic acid inhibited platelet adhesion on a collagen-coated surface. Cytochalasin D was used as a positive control. These studies showed that platelets preincubated with cytochalasin D prevented filopodia production and actin polymerization as expected [16]. When platelets are dispersed on collagen, the peripheral rim of F-actin at the leading edges of the lamellae is noticeably absent. Moreover, there was a reduction in filopodia production and actin polymerization when platelets were preincubated with cytochalasin D. These observations suggest that the betulinic acid inhibits SYK phosphorylation, resulting in inhibition of cytoskeletal reorganization, which in turn results in inhibition of platelets.

As this is an in vitro study, further studies will be carried out in animal models to assess its efficacy. Additionally, the effect of betulinic acid on SYK phosphorylation upon activation with various other platelet agonists, such as U46619, ADP, thrombin, and PMA, needs to be investigated in the future.

## Conclusions

These studies confirm the antiplatelet activities of betulinic acid and delve into the effect of betulinic acid on SYK phosphorylation. These studies demonstrate that betulinic acid inhibits collagen-induced platelet activation and platelet adhesion through inhibition of SYK phosphorylation. There are many signaling pathways involved in intracellular signal transduction, such as MAPK, ERK, and PKC, which also need to be investigated to determine whether the signaling pathways mediated by them are affected upon betulinic acid treatment. Further, various in vivo studies need to be carried out to assess the overall anti-platelet and antithrombotic activity of betulinic acid in comparison to the existing anti-platelet therapeutics such as aspirin, clopidogrel, ticlopidine, etc.
